# A Case of Ileus Relieved by Tobacco

**Published:** 1846-05

**Authors:** T. W. Fry

**Affiliations:** Crawfordsville, Indiana


### FACTS FROM CORRESPONDENTS.

---

### A CASE OF ILEUS RELIEVED BY TOBACCO.

By T. W. Fry, M.D., of Crawfordsville, Indiana.

Mrs. Winters, 69 years of age, suffered from a severe attack of intermittent fever last fall, and continued delicate and feeble through the winter. On the 16th of March she was seized with symptoms of colic, accompanied by constaut efforts at vomiting. A potion of tinct. opii and sulph. ether gave but temporary relief. On the morning of the 17th vomiting returned, and some bilious matter was thrown up; an emetic of ipecacuanha was now ordered, which relieved the stomach of a large quantity of bile. The sickness and pain having subsided to some extent, cathartic medicines, followed by saline mixtures, were had recourse to, but without any good effect. The pain returning with greater violence, the bowels becoming more swollen, accompanied by copious and frequent discharges of feculent matter from the mouth, now indicated more clearly the nature of the difficulty; and as all medicine taken into the stomach for the purpose of acting on the bowels only increased the sickness, pain, and vomiting, reliance was placed entirely upon opiates and injections. Tinct. opii 30 drops was given, followed in fifteen minutes by one-fourth of a grain of sulph. morphine; injections of salt water and castor oil frequently repeated, were immediately expelled without producing any action on the bowels, except one small discharge of opaque mucus. An injection of tobacco was now given with the same result. The bowels seeming incapable of retaining fluid matter, a suppository made in the following manner, was introduced into the rectum: a drachm of strong tobacco steeped in a gill of hot water was added to two tablespoonsful of sugar, and boiled to the consistence of wax. This was retained several hours, and the patient beginning to feel an inclination to stool, another injection was administered, which came away with a copious discharge from the bowels, and entire relief to the patient.

The discharge of feculent matter by the mouth, which continued up to the time of the introduction of the suppository, now ceased, and the patient, although prostrated to an alarming extent, her constitution being much impaired by age, recovered with great rapidity. Counter-irritation on the abdomen was kept up through the whole course of the disease by means of hot salt and mustard alternately applied.

The progress of this case, and of one terminating fatally which came under my observation last spring, convinces me that aperients in severe cases of ileus are only productive of evil.

March 27th, 1846.

